# Gβ-Like CpcB Plays a Crucial Role for Growth and Development of *Aspergillus nidulans* and *Aspergillus fumigatus*

**DOI:** 10.1371/journal.pone.0070355

**Published:** 2013-07-30

**Authors:** Qing Kong, Long Wang, Zengran Liu, Nak-Jung Kwon, Sun Chang Kim, Jae-Hyuk Yu

**Affiliations:** 1 School of Food Science and Engineering, Ocean University of China, Qingdao, Shandong, People’s Republic of China; 2 Departments of Bacteriology and Genetics, University of Wisconsin, Madison, Wisconsin, United States of America; 3 Systematic Mycology & Lichenology Lab, Institute of Microbiology, Chinese Academy of Sciences, Beijing, People’s Republic of China; 4 College of Bioscience & Bioengineering, Hebei University of Economics and Business, Shijiazhuang, Hebei, People’s Republic of China; 5 Department of Biological Sciences, Korea Advanced Institute of Science and Technology (KAIST), Dae-Jon, Republic of Korea; Universidade de Sao Paulo, Brazil

## Abstract

Growth, development, virulence and secondary metabolism in fungi are governed by heterotrimeric G proteins (G proteins). A Gβ-like protein called Gib2 has been shown to function as an atypical Gβ in Gpa1-cAMP signaling in *Cryptococcus neoformans*. We found that the previously reported CpcB (cross pathway control B) protein is the ortholog of Gib2 in *Aspergillus nidulans* and *Aspergillus fumigatus*. In this report, we further characterize the roles of CpcB in governing growth, development and toxigenesis in the two *aspergilli*. The deletion of *cpcB* results in severely impaired cellular growth, delayed spore germination, and defective asexual sporulation (conidiation) in both *aspergilli*. Moreover, CpcB is necessary for proper expression of the key developmental activator *brlA* during initiation and progression of conidiation in *A. nidulans* and *A. fumigatus*. Somewhat in accordance with the previous study, the absence of *cpcB* results in the formation of fewer, but not micro-, cleistothecia in *A. nidulans* in the presence of wild type *veA*, an essential activator of sexual development. However, the *cpcB* deletion mutant cleistothecia contain no ascospores, validating that CpcB is required for progression and completion of sexual fruiting including ascosporogenesis. Furthermore, unlike the canonical GβSfaD, CpcB is not needed for the biosynthesis of the mycotoxin sterigmatocystin (ST) as the *cpcB* null mutant produced reduced amount of ST with unaltered STC gene expression. However, in *A. fumigatus*, the deletion of *cpcB* results in the blockage of gliotoxin (GT) production. Further genetic analyses in *A. nidulans* indicate that CpcB may play a central role in vegetative growth, which might be independent of FadA- and GanB-mediated signaling. A speculative model summarizing the roles of CpcB in conjunction with SfaD in *A. nidulans* is presented.

## Introduction

Components of a heterotrimeric G protein (G protein) including FadA (Gα), GanB (Gα), SfaD (Gβ) and GpgA (Gγ) govern spore germination, vegetative growth, development, stress response, and toxigenesis in the model filamentous ascomycete *Aspergillus nidulans* [1]. Genetic studies have revealed that activated FadA-GTP transduces signals in part through a cAMP-dependent protein kinase (PkaA), leading to stimulation of vegetative proliferation, inhibition of sexual and asexual development, and suppression of biosynthesis of the carcinogenic mycotoxin sterigmatocystin (ST), the penultimate precursor of the better-known aflatoxins [2]–[4]. FlbA is an RGS (regulator of G protein signaling) protein that negatively regulates FadA-mediated growth signaling likely by enhancing the intrinsic GTPase activity of FadA [5], [6]. Loss of *flbA* function and FadA dominant-activating mutations cause uncontrolled proliferation of undifferentiated vegetative cell mass followed by colony autolysis. The null or dominant interfering mutant alleles of *fadA* bypass the need for FlbA in conidiation and ST production [2], [7]. RgsA is a specific RGS protein that primarily attenuates GanB signaling [8]. GanB-mediated signaling stimulates spore germination and stress responses, and inhibits asexual sporulation (conidiation) in part through PkaA. The *rgsA* deletion mutant exhibited a phenotype highly similar to that of the GanB^d+^ (Q208L) mutant [9], [10], i.e. reduced colony size, elevated germination without external carbon sources and accumulation of dark brown pigments. Conversely, the deletion of *ganB* suppresses the phenotypes caused by Δ*rgsA* [8].”

The *A. nidulans* Gβγ subunits play an equally important role in governing growth and development. The canonical Gβ subunit SfaD is composed of 352 amino acids sharing ∼60% identity with mammalian Gβ, and has a conserved Trp-Asp sequence referred to as the “WD-40” motif [11]. The single Gγ subunit GpgA consists of 90 amino acids showing 72% similarity with the yeast Ste18p [12]. GpgA contains a typical coiled-coil domain at the N-terminal region, which is necessary for the interaction of a Gγ with a cognate Gβ to form a heterodimer [13]. The deletion of *sfaD* or *gpgA* results in highly restricted vegetative growth and rescues certain conidiation defects caused by the absence of *flbA*, providing a key genetic evidence that SfaD and GpgA function in the FadA-mediated vegetative growth signaling. The SfaD:GpgA heterodimer also functions with GanB, and GanB and SfaD:GpgA are associated with spore germination, carbon source sensing, stress responses and hyphal pigmentation in *A*. *nidulans* [11], [13], [14]. Because the deletion of either *sfaD* or *gpgA* results in the lack of cleistothecia (sexual fruiting bodies) in self-fertilization and causes a severe impairment in sexual development in outcrosses, it has been proposed that SfaD and GpgA constitute the primary signaling component for sexual reproduction in *A. nidulans* [1], [11], [13], [14].

The opportunistic human pathogen *Aspergillus fumigatus* causes mycosis, allergy, and invasive aspergillosis in immune-compromised individuals [15]. As G protein components are highly conserved in eukaryotes, the corresponding *A. fumigates* orthologues of the above mentioned *A. nidulans* G protein components show high levels of identity [16]–[18]. In our previous study, we demonstrated that the FadA homologue GpaA mediates signaling for vegetative growth, which in turn negatively controls conidiation, and that GpaA signaling is attenuated by *Afu*FlbA [1], [18]–[20]. We also characterized the homologues of SfaD and GpgA in *A. fumigatus* and presented a series of evidence that *Afu*SfaD and *Afu*GpgA play a crucial role in governing vegetative growth, spore germination, conidiation and production of certain secondary metabolites [21].

A novel Gβ-like protein, Gib2, has been identified in the human pathogenic fungus *Cryptococcus neoformans* as an interacting protein of the Gα subunit Gpa1 in a yeast two-hybrid screen [22]. Gib2 is a homolog of the Gβ-like/receptor for activated protein kinase C1 (RACK1), which had originally been identified as a putative intracellular receptor for activated protein kinase C [23]. *C. neoformans* Gib2 contains a seven WD-40 repeat motif and is predicted to form a seven-bladed β propeller structure that is characteristic of β transducins. It was further demonstrated that Gib2 functions as an atypical Gβ in Gpa1-cAMP signaling that likely stabilizes Gpa1 and facilitates its oscillation between ON and OFF status, and thereby regulates cAMP signaling. Gib2 was shown to physically interact with the two Gγ subunits Gpg1 and Gpg2, similar to the conventional Gβ subunit Gpb1. Knock-down of *gib2* by antisense suppression resulted in a severe growth defect, indicating that Gib2 plays a key role in promoting vegetative proliferation in *C. neoformans* [22]. These studies suggest that a Gib2 ortholog might play an important role in *Aspergillus* proliferation, where three Gα subunits but only one Gβ subunit has been identified.

Our search for a potential ortholog of Gib2 has identified CpcB in the *A. nidulans* genome. CpcB was previously identified and characterized as a component of “cross pathway control” [24]. Amino acid limitation results in impaired sexual fruiting in *A. nidulans*. Hoffman et al [24] revealed that the c-Jun homolog CpcA and the RACK1 homolog CpcB coordinate sexual developmental progression illuminating a possible connection point between metabolism and sexual development. Amino acid starvations activate the cross-pathway regulatory network including the transcriptional activator CpcA, leading to induction of a number of amino acid biosynthetic genes. CpcB negatively regulates the cross-pathway network in the presence of amino acids. When *A. nidulans* is grown under amino acid starvation conditions, the fungus is unable to complete meiosis and sexual fruiting, producing microcleistothecia filled with hyphae. Addition of amino acids removes this specific developmental block and leads to completing sexual fruiting including the development of mature ascospores. Hoffman et al [24] demonstrated that the identical developmental block is induced by either overexpression of a c-Jun homolog (*cpcA*, GCN-4, or cpc-1) or deletion of *cpcB* in the presence of amino acids. Furthermore, the deletion of *cpcB* resulted in the increased expression of amino acid biosynthetic gene (*argB*). Collectively, CpcA (activated by aa starvation) inhibits the progression of sexual fruiting before meiosis, and CpcB (represses aa biosynthesis in the presence of aa) confers the progression and completion of sexual development including maturation of ascospores.

In *Aspergillus*, asexual development (conidiation) results in the formation of a massive number of conidia that form on the specialized structures called conidiophores [25]–[27]. A key and essential step for conidiophore development is activation of *brlA*, which encodes a C_2_H_2_ zinc finger transcription factor activating conidiation specific genes and *abaA* [28], [29]. The *abaA* gene encodes a developmental regulator that is activated during the middle stages of conidiophore development after metulae differentiation [30], [31]. The *wetA* gene functions in late phase of conidiation for the synthesis of crucial cell wall components [32], [33]. These three genes have been proposed to define a central regulatory pathway that acts in concert with other genes to control conidiation-specific gene expression and determine the order of gene activation during conidiophore development and spore maturation [25], [26], [34]. VosA is a *velvet*-domain protein that exerts feedback regulation of *brlA* and couples sporogenesis and trehalose biogenesis in spores, required for the long-term viability of conidia and ascospores, thereby completing conidiogenesis [35].

While CpcB’s functions in cross-pathway control and sexual fruiting have been characterized, its role in governing spore germination, vegetative growth, and asexual development (conidiation) in *Aspergillus* has remained to be investigated. Moreover, Hoffman et al. [24] examined the role of CpcB in sexual development using a lab strain with the *veA1* allele [36], which lacks the full potential for sexual development. VeA is the founding member of the fungi-specific *velvet* proteins, and it forms the VelB-VeA and/or VelB-VeA-LaeA complexes that control the initiation and progression of sexual fruiting and secondary metabolism [36]–[39]. Therefore, in order to further assess the precise role of *cpcB* in sexual development, we have characterized the *cpcB* null mutation with the wild type (WT) *veA* allele. Here, we report the roles of CpcB in governing growth, asexual/sexual development and toxigenesis in both *A. nidulans* and *A. fumigatus*.

## Materials and Methods

### *Aspergillus* Strains and Culture Conditions

*Aspergillus* strains used in this study are listed in Table 1. Fungal strains were grown on solid or liquid minimal glucose medium with appropriate supplements (e.g. 1 g uracil l^−1^+1 g uridine l^−1^, or 1 mL 1% pyridoxine per liter; simplified as MM), or with yeast extract (YE) as described [40], and incubated at 37°C. *Escherichia coli* DH5α was used for routine cloning of constructs and cultured in LB broth, Miller (Novagen, CA), at 37°C and supplemented with appropriate antibiotics [41].

**Table 1:** *Aspergillus* strains used in this study.

Strain	Genotype	Source
***A. nidulans***		
FGSC4	Wild Type (*veA* ^+^)	FGSC^a^
RJMP 1.59	*pyrG89*; *pyroA4*; *veA* ^+^	[60]
TNJ36	*pyrG89*; *pyroA4*; *AfupyrG* ^+^; *veA* ^+^ (control strain)	[61]
RJMP1.59-8^b^	*pyrG89*; *pyroA4*; Δ*cpcB*::*AfupyrG* ^+^; *veA* ^+^	This study
RJMP1.59-8C^b^	*pyrG89; pyroA4*; Δ*cpcB*::*AfupyrG* ^+^; *AnicpcB*(p)::*AnicpcB^+^*; *veA* ^+^	This study
TNJ36.1	*pyrG89*; *pyroA4*; *pyrG* ^+^; *veA* ^+^	This study
QK1^b^	*pyrG89*; *pyroA4*; *pyrG* ^+^; Δ*flbA*::*AnipyroA* ^+^; *veA* ^+^	This study
QK2^b^	*pyrG89*; *pyroA4*; *pyrG* ^+^; Δ*rgsA*::*AnipyroA* ^+^; *veA* ^+^	This study
QK3^b^	*pyrG89*, Δ*cpcB*::*AfupyrG* ^+^; *pyroA4*, Δ*flbA*::*AnipyroA* ^+^; *veA* ^+^	This study
QK4^b^	*pyrG89*, Δ*cpcB*::*AfupyrG* ^+^; *pyroA4*, Δ*rgsA*::*AnipyroA* ^+^; *veA* ^+^	This study
***A. fumigatus***		
Af293	Wild Type	[62]
Af293.1	*AfupyrG1*	[63]
Af293.1-7^b^	*AfupyrG1*; Δ*cpcB*::*AnpyrG* ^+^	This study
Af293.1-7C^b^	*AfupyrG1*; Δ*cpcB*::*AnpyrG* ^+^; *AfucpcB*(p)::*AfucpcB* ^+^	This study

a Fungal Genetics Stock Center.

b Multiple isogenic strains (all behaved identically).

For phenotypic analyses of *Aspergillus* strains on air-exposed culture, conidia (∼10^4^) of relevant strains were spotted in 2-µl aliquots on appropriate solid MM and incubated at 37°C for 4 days. Conidia were collected in 0.1% Tween 80 from the entire colony and counted using a hemacytometer. To examine development and secondary metabolite production in liquid submerged culture, spores of relevant strains were inoculated to a final concentration of 10^6^ conidia/ml in 100 ml of liquid MM, or with YE and incubated at 220 rpm at 37°C. For induction of asexual (and sexual) development, the conidia (10^6/^ml) of WT and mutant strains were inoculated in 300 ml of liquid MM, or with YE in 1-liter flasks and incubated at 37°C, 220 rpm for 14 to 18 h ( = 0-h time point for developmental induction). The mycelium was harvested by filtering through Miracloth (Calbiochem, CA) and transferred to solid MM and incubated at 37°C for air-exposed asexual developmental induction or tightly sealed from air and light for sexual developmental induction as described [42]. To study sexual developmental induction in *A. nidulans*, sexual induction medium with appropriate supplements (SMG) was used. When appropriate, ten cleistothecia were selected from individual strains to compare the shape of cleistothecia and the number of ascospores. The plates and mycelium pellets of relevant strains were visually and microscopically examined. Samples collected at various time points of the liquid submerged culture and post-asexual developmental induction were placed in-between the paper-towel, squeezed to remove excess medium, transferred to a micro-centrifuge tube and stored at −80°C until subjected to total RNA isolation. For the determination of germination rates and the length of hyphae, about 10^6^ conidia of relevant strains were spread onto solid MM. Cultures were grown at 37°C and the spores were considered germinated when presenting a protrusion corresponding to the emerging germ tube. Three sets of 10 spores were monitored at each time point [10].

### Construction of *A. nidulans* Strains

The oligonucleotides used in this study are listed in Table S1. For the deletion of *cpcB*, double-joint PCR (DJ-PCR) method was used [43]. Both flanking regions of *cpcB* were amplified using the primer pairs oPX-105/oPX-106 and oPX-107/oPX-108 and using FGSC4 genomic DNA as a template. The *A. fumigates pyrG* marker was PCR-amplified from AF293 genomic DNA with the primer pair oJH-83/oJH-86. The three fragments were fused, and the final PCR product for the *cpcB* deletion was amplified using the primer pair oPX-109/oPX-110, and introduced into RJMP 1.59 protoplasts generated by the Vinoflow FCE lysing enzyme (Novo Nordisk) [44]. The Δ*AnicpcB* mutants (RJMP1.59-8) were isolated, and confirmed by PCR and subsequent restriction enzyme digestion of the amplicon [43]. At least three independent deletion strains were isolated. Likewise, 5′ and 3′ flanking regions of *flbA* (oNK-412/oNK-413 and oNK-414/oNK-415) and*rgsA* (oNK-540/oNK-541 and oNK-542/oNK-543) were amplified, fused with the *A. nidulans pyroA* (amplified with oNK-395/oNK-396) selective marker by DJ-PCR, and used to make the final PCR amplicons (oNK-416/oNK-417 and oNK-544/oNK-545). Each construct for the deletion of *flbA* or *rgsA* was introduced into TNJ36.1 to acquire the Δ*AniflbA* (QK1) and Δ*AnirgsA* (QK2) mutants, or introduced into RJMP1.59-8 to acquire the double deletion mutants (QK3 and QK4).

To complement Δ*AnicpcB*, the WT *AnicpcB* gene (3673bp) was amplified from FGSC4 by oLW-1 and oLW-1. The *Kpn*I and *Eco*RI digested *AnicpcB* amplicon was cloned into pHS8 and the resulting recombinant DNA was introduced into *E. coli*. Individual clones were sequence verified the final plasmid pLW1 was used to transform RJMP1.59-8 into the complemented strain RJMP1.59-8C, verified by oHS-350 and oHS-351.

### Construction of *A. fumigatus* Strains

The *AfucpcB* gene was deleted in the *A. fumigatus* AF293.1 (*pyrG1*) strain employing DJ-PCR [43]. The 5′ and 3′ flanking regions of the *AfucpcB* gene was amplified from *A. fumigatus* AF293 genomic DNA with the primer pairsoPX-111/oPX-112 and oPX-113/oPX-114. The *A. nidulans pyrG^+^* marker was amplified from FGSC4 genomic DNA with the primer pair oBS-08/oBS-09. The 5′ and 3′ flanking regions of *AfucpcB* were fused to the marker and further amplified by the nested primer pair oPX-115/oPX-116, yielding the final *AfucpcB* gene deletion construct. The gene deletion construct was introduced into the recipient strain AF293.1, and the Δ*AfucpcB* mutants (e.g., Af293.1-7) were isolated and confirmed by PCR followed by restriction enzyme digestion [43]. At least three independent deletion strains were isolated.

To complement Δ*AfucpcB*, the hygromycin resistance gene from plasmid pUCH2-8 and the *AfucpcB* gene from wild-type AF293 strain were amplified by primer pairs oLW-3/oLW-9 and oLW-10/oPX-114, respectively. Fusing them by single joint PCR with the primer pair oLW-4/oLW-7 resulted in the final 5.8 kb complementation construct, which was then used to transform Afu293.1-7 (Δ*AfucpcB*), and transformants resistant to 100 µg/ml hygromycin were isolated. The primer pair oLW-3/oLW-11 was used to check the presence of the WT *cpcB* allele in the transformants, leading to isolation of the complemented strain Af293.1-7C.

### Nucleic Acid Isolation and Manipulation

Genomic DNA isolation, total RNA isolation and Northern blot analyses were carried out as described previously [19], [43], [45]. Total RNA was isolated from conidia and mycelia collected at various time points. All samples were squeeze-dried, quick-frozen in liquid N_2_ and stored at −80°C until subjected to RNA isolation. Approximately 10 µg (per lane) of total RNA isolated from individual samples was separated by electrophoresis using a 1% agarose gel containing 3% formaldehyde and ethidium bromide and blotted onto a Hybond-N membrane (Amersham, NY). The [^32^P] dCTP-labeled hybridization probes for *AnibrlA*, *AniabaA*, *AniwetA*, *AnivosA*, *AninsdD*, *AniveA*, *AnistcU*, *AniaflR*, *AfubrlA*, *AfuabaA* and *AfuwetA* were prepared by PCR amplification of individual ORFs from the genomic DNA of FGSC4 and AF293 by using specific oligonucleotides (Table S1; Fig. S1).

### ST and GT Analysis

Spores (∼10^6^) of each strain were inoculated into 3 ml liquid MM with 0.5% YE in 8-ml tubes and the stationary cultures were incubated at 37°C for 1, 2, 4, 6, 8 days, respectively, as described [46]. ST was extracted from 1, 2, 4, 6, 8-day-old cultures by adding 1 ml of CHCl_3_ to each tube and then vortexing for 1 min. The organic phase was transferred to 1.5 ml tubes and centrifuged at 500×g for 5 min at 4°C. The CHCl_3_ layer was collected, dried, and resuspended in 50 µl of CHCl_3_ and 10 µl of each sample was applied onto a thin layer chromatography (TLC) silica plate containing a fluorescence indicator (Kiesel gel 60, 20 cm×20 cm, 0.25 mm thick; E. Merck). ST standard was purchased from Sigma and about 5 µg was applied onto the TLC plate with the samples. The plate was then developed with toluene:ethylacetate:acetic acid (80∶10:10, v/v/v), where the R_f_ value of ST is about 0.65. At this step ST exhibits dark-red color under the long wave UV (320 nm). To enhance visibility and the detection limit of ST, aluminum chloride (20% AlCl_3_^.^6H_2_O in 95% ethanol) is sprayed on to the TLC plate and the plate is baked at 80°C for 5 min. The color of ST changes from red to bright light-green by this process [47]. Photographs of TLC plates were taken following exposure to UV radiation using a Sony DSC-F828 digital camera.

To assess the production of GT, conidia of each strain were inoculated into 50 ml liquid YM and incubated for 2 days at 37°C and 250 rpm. GT was extracted with chloroform as described previously [48]. The chloroform extracts were air-dried and resuspended in 100 µl of methanol. Ten µl of each sample was applied to a thin-layer chromatography (TLC) silica plate containing a fluorescence indicator (Kiesel gel 60, E. Merck). TLC plate was developed with chloroform:acetone (7∶3, v/v) until the solvent front reached about 15 cm. GT standard was purchased from Sigma.

### Microscopy

Photomicrographs were taken by using a Zeiss Axioplan 2 microscope with AxioVision digital imaging software (Zeiss). Culture plate and Northern blot photographs were taken using a SONY DSC-F828 digital camera.

### Statistical Analysis

All experiments were performed triplicates for each condition analyzed. Data are shown as mean±SD and bars indicate SD. Statistical differences between WT and mutant strains were evaluated with Student’s unpaired t-test. P values <0.05 were considered significant.

## Results

### Gene Structure and Expression of *cpcB*

**Figure 1:**
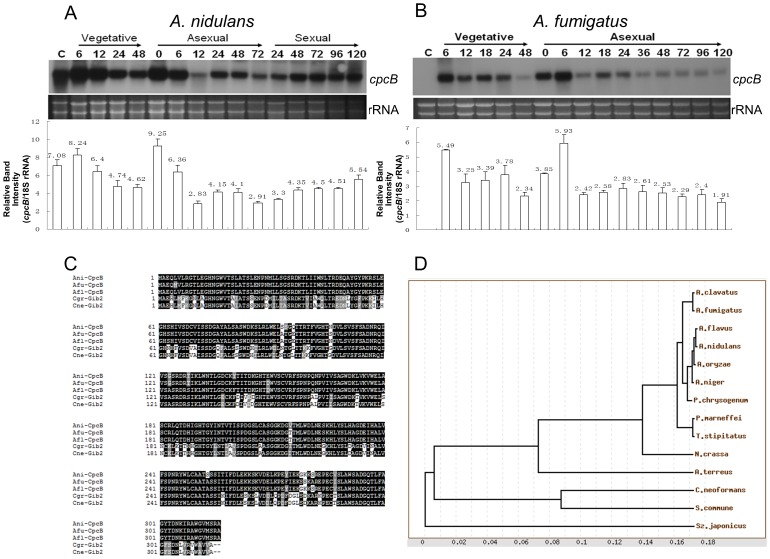
Summary of *cpcB*. (A∼B) *cpcB* mRNA levels during the life cycle of *A. nidulans* (A) and *A. fumigatus* (B). Conidia (asexual spores) are indicated as C. The numbers indicate the time (hours) after incubation in liquid MMG (Vegetative), and on solid MMG under conditions inducing conidiation (Asexual) or sexual development (Sexual). The relative band intensities (mean±SD) of Northern blot were determined by densitometric scanning of mRNA bands using ImageJ and each band was normalized by the amount of 18S rRNA (*cpcB*/18S rRNA) in the relevant lane. Ethidium bromide staining of ribosomal RNAs was used as a loading control. (C) Alignment of *A. nidulans* (Ani) CpcB, *A. fumigatus* (Afu) CpcB, *A. flavus* (Afl) CpcB with Gib2 of *C. neoformans var. grubii* (Cgr; accession: AY907679) and *C. neoformans var. neoformans* (Cne; accession: AY907680). ClustalW (http://align.genome.jp/) and BoxShade 3.21 (http://www.ch.embnet.org/software/BOX_form.html) were used for the alignment and presentation. (D) A phylogenetic tree of CpcB-like proteins identified in various fungal species including *A. clavatus*, *A. flavus*, *A. fumigatus*, *A. nidulans*, *A. niger*, *A. oryzae*, *A. terreus (A = Aspergillus)*, *C. neoformans*, *Neurospora crassa*, *Penicillium chrysogenum*, *Penicillium marneffei*, *Schizophyllum commune*, *Schizosaccharomyces japonicus* and *Talaromyces stipitatus*. The putative CpcB proteins were retrieved from NCBI BlastX (http://blast.ncbi.nlm.nih.gov/Blast.cgi) using *A. nidulans* CpcB. A phylogenetic tree of 14 putative CpcB homologues was generated by the TreeTop software (http://genebee.msu.su/services/phtree_reduced.html) using the alignment data from ClustalW. The phylogenetic tree is constructed based on the matrix of pairwise distances between sequences. Numbers indicate the computed distances given the residue substitution weights from the alignment data [59].

BLAST searches of the *A. nidulans* and *A. fumigatus* genome (AspGD) employing the *C. neoformans* Gib2 protein [22] led to the identification of the *cpcB* gene in *A. nidulans* [24] and *A. fumigatus*. The *cpcB* gene maps to chromosome II in *A. nidulans* and the ORF consists of 1,430 bp, interrupted by three introns (144 bp, 128 bp and 213 bp), and is predicted to encode a 316 aa-length protein (AN4163; AF176775). The *A. fumigates cpcB* gene maps to chromosome IV, and its ORF consists of 1447 bp, interrupted by three introns (142 bp, 125 bp and 235 bp), and is predicted to encode a 316 aa-length protein (Afu4g13170). *cpcB* mRNA accumulates at varying levels throughout the lifecycle in both species. In *A. nidulans*, levels of *cpcB* mRNA are high in conidia and vegetative cells, and moderate in the asexual and sexual development phases (Fig. 1A). In *A. fumigatus*, *cpcB* mRNA is detectable at 6 h of vegetative growth, maintained at high levels during the early phases (0–6 h) of asexual development, reduced in the late phase (72–120 h) of conidiation, and absent in conidia (Fig. 1B). Protein database searches revealed that proteins similar to CpcB are present in many other filamentous Ascomycetes and all *aspergilli* examined (Fig. 1C and 1D). Amino acid sequence of CpcB (or Gib2) is highly conserved in *A. nidulans*, *A. fumigatus*, *A. flavus*, *C. neoformans var. grubii* (accession: AY907679) and *C. neoformans var. neoformans* (accession: AY907680).

### Requirement of CpcB for Proper Growth and Conidiation

**Figure 2:**
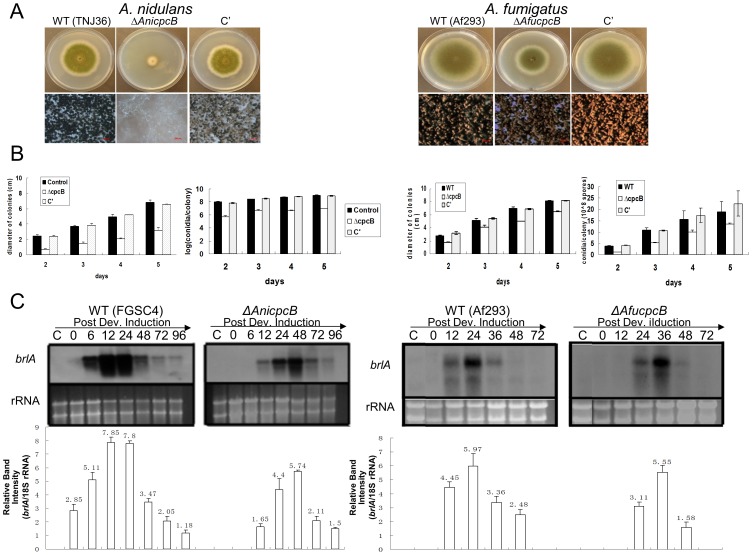
Requirement of CpcB for proper growth and conidiation in *A. nidulans* and *A. fumigatus*. (A) Photographs of the point-inoculated colonies of WT control (TNJ36), Δ*cpcB* (RJMP1.59-8) and complemented (C’; RJMP1.59-8C) strains of *A. nidulans* grown on solid MMG for 4 days (top panels) and the close-up views of the colonies (bottom panels) are shown in left panel. Photographs of the colonies of WT (AF293), Δ*AfucpcB* (Af293.1-7) and complemented (C’; Af293.1-7C) strains of *A. fumigatus* grown on solid MM+0.5%YE for 4 days (top panels), and the close-up views of the colonies (bottom panels) are shown in right panel. (B) Quantitative analyses of the diameter of colonies and conidiation levels in the designated strains. (C) Northern blot analyses for levels of *brl*A transcript in the WT (FGSC4) and Δ*AnicpcB* (RJMP1.59-8) strains of *A. nidulans*, and in WT (Af293) and Δ*AfucpcB* (Af293.1-7) strains of *A. fumigatus.* The relative band intensities (mean±SD) of Northern blot were determined by densitometric scanning of mRNA bands using ImageJ and normalized by the amounts of 18S rRNA (*brlA*/18S rRNA) in each sample. Ethidium bromide staining of rRNA was used as a loading control.

We generated individual null mutants lacking *AnicpcB* (Δ*AnicpcB*; RJMP1.59-8) and *AfucpcB* (Δ*AfucpcB*; Af293.1-7) by replacing its coding region with *AfupyrG* ^+^ and *AnipyrG* ^+^, respectively (see Table 1). Subsequently, using each deletion strain as a transformation host, we further generated complemented strains (C’) by introducing the WT *AnicpcB* allele into the *pyroA* locus, and the WT *AfucpcB* allele linked with HygR, respectively. We then examined the phenotypes of WT, Δ*cpcB* and complemented strains (C’; Table 1). Most markedly, as shown in Fig. 2A, the deletion of *cpcB* resulted in highly restricted vegetative growth with nearly absent (in case of *A. nidulans*) or reduced (in case of *A. fumigatus*) conidiation. Quantitative analyses of conidia per colony grown on solid medium further demonstrated that asexual spore production in the Δ*cpcB* mutant was significantly reduced (*p*<0.01); approximately 1% of WT and C’ strains of *A. nidulans* and ∼30% of WT and C’ strains of *A. fumigates* (Fig. 2B). These results suggest that *cpcB* is necessary for proper proliferation and asexual development in air-exposed solid cultures of *A. nidulans* and *A. fumigatus*.

We further examined the effects of Δ*cpcB* during the progression of conidiation upon developmental induction. To minimize the effects of growth defects on expression of *brlA*, the same amount of wet mycelia of WT and the mutants was transferred onto the solid medium, and the equal amount of the developing cells was subject to RNA analyses. During vegetative growth of *A. nidulans*, no differences in mRNA levels of *brlA*, *abaA*, *wetA* and *vosA* between WT and the null mutant were observable (data not shown). Upon induction of asexual development, however, WT strain began to accumulate *brlA* mRNA at 6 h, with a peak at 12 h postinduction, whereas the Δ*cpcB* mutant started to express *brlA* at 12 h, with a peak at 48 h, i.e., 12-36 h delay (Fig. 2C). Likewise, WT strain began accumulating *abaA*, *wetA* and *vosA* mRNA at 12 h, with a peak at 12-24 h post-induction, whereas the Δ*cpcB* mutant exhibited highly reduced and delayed accumulation of *abaA*, *wetA* and *vosA* transcripts during the progression of conidiation (see Fig. S1). These results indicate that CpcB is necessary for proper expression of *brlA*, *abaA*, *wetA* and *vosA* during the initiation and progression of conidiation in *A. nidulans*.

In *A. fumigatus*, upon induction of asexual developmental, WT strain accumulated *AfubrlA* mRNA at 12 h, with a peak at 24 h post-induction, whereas the Δ*AfucpcB* mutant started to accumulate *AfubrlA* at 24 h, with a peak at 36 h (Fig. 2C). Similarly, WT strain began accumulating *AfuabaA* mRNA at 24 h, while the Δ*AfucpcB* mutant started to express *AfuabaA* at 36 h; i.e., about a 12 h delay in *AfubrlA* and *AfuabaA* expression (data not shown). These results indicate that *Afu*CpcB is necessary for proper expression of *Afubrl*A and *Afuaba*A during the initiation and progression of conidiation in *A. fumigatus*, too.

### CpcB is Necessary for Sexual Development in *A. nidulans*

**Figure 3:**
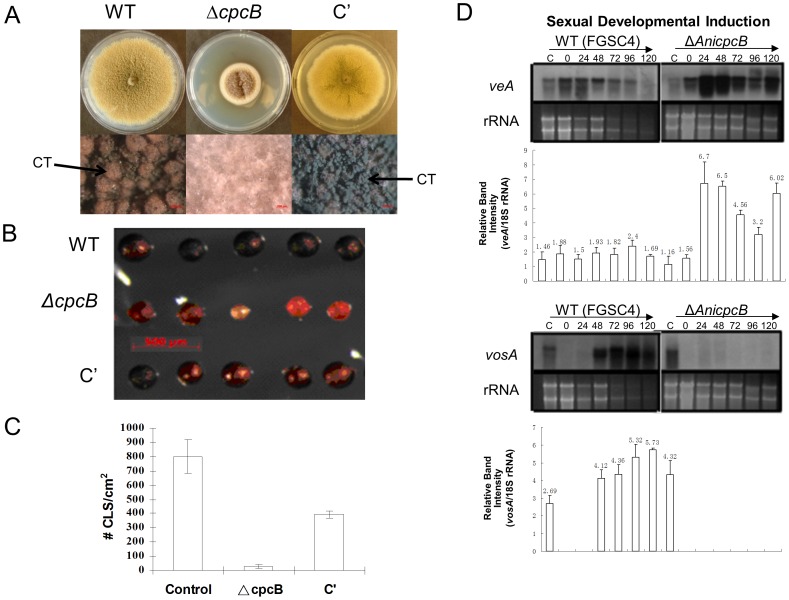
Requirement of CpcB for sexual development in *A. nidulans*. (A) Photographs of the point-inoculated colonies of WT (TNJ36), Δ*cpcB* (RJMP1.59-8) and complemented (C’; RJMP1.59-8C) strains of *A. nidulans* grown on MMG for 7 days (top panels), and the close-up views of the colonies (bottom panels) captured by a Zeiss Axioplan 2 stereomicroscope are shown. Cleistothecia are marked as CT. (B) Morphology of cleistothecia formed by the three strains. Images were captured by a Zeiss Axioplan 2 stereomicroscope. (C) Quantitative analyses of cleistothecia (CLS) per cm^2^ produced by the three strains grown on MMG for 7 days. (D) Northern blot analyses for levels of *veA* and *vos*A transcripts after sexual developmental induction of *A. nidulans* WT (FGSC4) and Δ*cpcB* (RJMP1.59-8) strains. The relative band intensities (mean±SD) of Northern blot were determined by densitometric scanning of RNA bands using ImageJ and normalized by the amounts of 18S rRNA (*veA*/18S rRNA or *vosA*/18S rRNA) in each sample. Ethidium bromide staining of ribosomal RNAs was used as a loading control. Numbers indicate the time post sexual developmental induction.

We further investigated the roles of CpcB in sexual fruiting of *A. nidulans* in the presence of the WT *veA* allele. When point-inoculated on MMG, the Δ*cpcB* mutant formed smaller, white colonies producing few cleistothecia (Fig. 3A), whereas WT and C’ strains formed cleistothecia abundantly. After incubating on MMG for 7 days, WT and C’ strains produced 800±117.85 and 394±24.58 cleistothecia/cm^2^, respectively. However, the Δ*cpcB* mutant produced 23.67±4.73 cleistothecia/cm^2^ and most (if not all) of cleistothecia were small, fragile and with reduced pigmentation (Fig. 3B and 3C). Furthermore, whereas WT and C’ cleistothecia contained 8.66(±1.82)×10^4^ and 5.7(±3.27)×10^4^ ascospores per cleistothecium, respectively, the Δ*cpcB* cleistothecia contained no ascospores. These results confirm that CpcB is required for proper formation of fruiting bodies and ascosporogenesis [24]. We noted that the presence of the WT *cpcB* allele in the *pyroA* locus appears to be insufficient to fully restore sexual fruiting of the Δ*cpcB* mutant to WT level. This might be due to the positional effects and/or the lack of key *cis* acting elements in the cloned *cpcB* WT gene region in the complementation construct, leading to improper expression of *cpcB in trans*.

We then examined mRNA levels of *veA* and *vosA* during the progression of sexual development employing homogeneous developmental induction. As shown in Fig. 3D, WT strain began accumulating *veA* mRNA at 0 h, with a peak at 24 h post induction, while the Δ*cpcB* mutant exhibited a very high level of *veA* mRNA at 0 h, with a peak at 24-48 h and 120 h, suggesting that CpcB is needed for proper down-regulation of *veA* during sexual development, which might be critical for proper progression of sexual fruiting. Another *velvet* regulator VosA is a key regulator of sporogenesis, which couples sporogenesis and focal trehalose biogenesis and is required for viable ascospore formation [35], [39]. Importantly, as shown in Fig. 3D, whereas WT strain began accumulating *vosA* mRNA at 48 h post induction of sexual development, the Δ*cpcB* mutant did not show any signs of *vosA* mRNA accumulation. These results indicate that CpcB plays an important role in controlling sexual development and expression of the *velvet* regulators, and that SfaD (the canonical Gβ) and CpcB (a Gβ-like protein) govern distinct stages of sexual fruiting in *A. nidulans* [11]. It has been proposed that SfaD (with GpgA) acts on the initiation of sexual fruiting, whereas CpcB functions during meiosis and ascosporogenesis [11], [13], [24].

### The Role of CpcB in Conidial Germination and Cellular Growth

**Figure 4:**
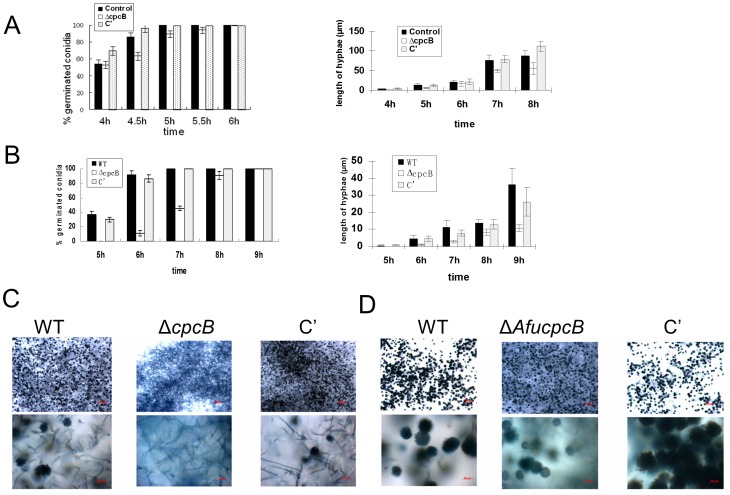
The roles of CpcB in conidial germination and hyphal growth. (A∼B) Quantitative analyses of conidial germination and hyphal growth post conidial germination of WT (filled bar; TNJ36), Δ*cpcB* (blank bar; RJMP1.59-8) and complemented (C) (shaded bar; RJMP1.59-8C) *A. nidulans* strains on MMG (A); and of WT (AF293), Δ*AfucpcB* (Af293.1-7) and complemented (C’; Af293.1-7C) *A. fumigates* strains on MMG (B). (C) Photographs of the three *A. nidulans* strains grown on solid MMG for 1 day (top panels) and the close-up views (bottom panels). Note the lack of conidiophores in Δ*cpcB* strain. (D) Photographs of the three *A. fumigates* strains of grown on solid MMG for 1 day (top panels), and the close-up views (bottom panels). Note the differences in the size and number of conidiophores.

The fact that *cpcB* mRNA is present abundantly in *A. nidulans* conidia, and accumulates to high levels during the entire phase of vegetative proliferation in both species (6 h, Fig. 1A and 1B) led us to examine whether the absence of CpcB would affect conidial germination and hyphal growth. As shown in Fig. 4A, WT *A. nidulans* conidia showed about 54% germination at 4 h, and 100% germination at 5 h. On the contrary, the Δ*cpcB* mutant conidia showed ∼53%∼60% germination rates at 4∼4.5 h, and 89% germination at 5 h (significantly delayed germination). The average length of hyphae of WT *A. nidulans* was 3.2±1.1 µm at 4 h, and 21.25±3.2 µm at 6 h, and those of Δ*cpcB* mutant was 1.7±0.57 µm at 4 h, and 16.4±6.11 µm at 6 h. As the *cpcB* mutant hyphae do not show differences in the width, these data suggest that CpcB required for proper cellular growth, and conidial germination in *A. nidulans*.

Likewise, a severe retardation of conidial germination and cellular growth was observed in the *A. fumigates cpcB* deletion mutant (Fig. 4B). WT *A. fumigates* conidia showed about 56% germination at 5 h and 100% germination at 7 h in MM. The average length of hyphae of WT *A. fumigates* strain was 4.63±2.21 µm at 6 h and 14±1.83 µm at 8 h. On the contrary, the Δ*AfucpcB* mutant conidia showed 0% germination at 5 h and ∼46% germination at 7 h in MM. The average hyphal length of the Δ*AfucpcB* mutant was 1.25±0.5 µm at 6 h and 8.25±1.71 µm at 8 h. In both species, after 1 day incubation on MMG WT and C’ strains produced a higher number of conidiophores than the *cpcB* deletion mutants (Fig. 4C and 4D). Collectively, these data indicate that CpcB plays a positive role in spore germination, cellular growth and conidiophore formation in both *Aspergillus* species.

### Role of CpcB in Mycotoxin Production

**Figure 5:**
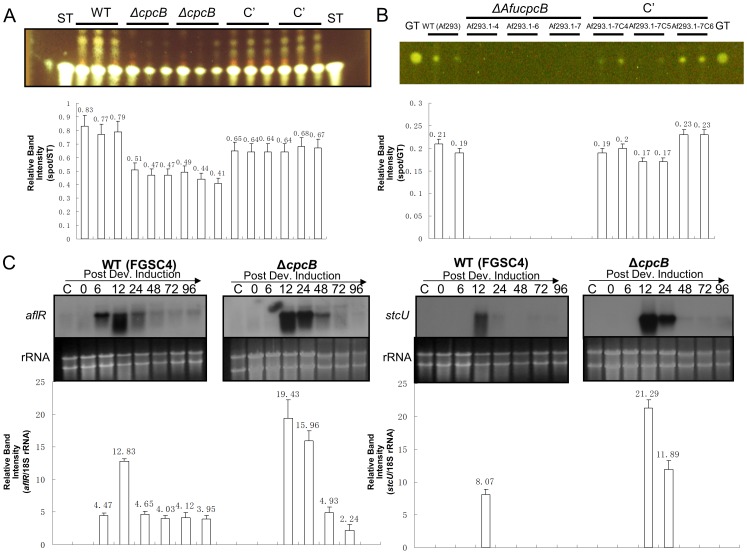
CpcB is not required for ST biosynthesis. (A) TLC of ST produced after 2 days of liquid submerged culture (MM+0.5%YE) of WT (FGSC4), Δ*cpcB* (RJMP1.59-8, RJMP1.59-24) and complemented (RJMP1.59-8C1, RJMP1.59-8C5) *A. nidulans* strains. The relative intensity (mean±SD) of ST produced by each strain was determined by ImageJ and normalized by the amount of the ST standard (spot/ST). (B) TLC of GT produced upon 2 days of liquid submerged culture (MM+0.5%YE) of WT (Af293), Δ*AfucpcB* (Af293.1-4, Af293.1-6, Af293.1-7) and complemented (Af293.1-7C4, Af293.1-7C5, Af293.1-7C6) *A. fumigatus* strains. The relative intensity (mean±SD) of GT produced by each strain was determined by ImageJ and normalized by the amount of the GT standard (spot/GT). (C) Northern blot for levels of *stcU* and *aflR* transcripts in WT (FGSC4) and Δ*cpcB* (RJMP1.59-8) of *A. nidulans*. The relative band intensities (mean±SD) of Northern blot were determined by densitometric scanning of mRNA bands using ImageJ and normalized by the amounts of 18S rRNA (*aflR*/18S rRNA or *stcU*/18S rRNA) in each sample. Ethidium bromide staining of ribosomal RNAs was used as a loading control.

Previously we showed that SfaD, GpgA and PhnA (a phosducin-like protein) are necessary for the expression of *aflR* encoding the transcriptional activator for the ST biosynthetic genes and subsequent ST biosynthesis [14]. We checked whether the absence of CpcB also affects ST biosynthesis in *A. nidulans*. As shown in Fig. 5A, compared to WT and C’ strains, two independent Δ*cpcB* mutant strains produced reduced amounts of ST and additional unknown compounds with higher Rf values (spots above ST). Production of ST requires coordinated expression of the pathway-specific transcription factor (AflR) and many biosynthetic enzymes including StcU [49]. We found that Δ*cpcB* did not affect mRNA accumulation of *aflR* and *stcU* (Fig. 5C; [49]) during asexual developmental induction. These results suggest that reduced production of ST by Δ*cpcB* is not caused by the lack of proper gene expression as observed in SfaD and PhnA mutant, but by an indirect effect [14].

*A*. *fumigatus* produces the non-ribosomal peptide toxin GT [50], which has immunosuppressive activity and contributes to the virulence of the fungus [51], [52]. Previously we showed that SfaD and GpgA are needed for the proper production of GT in *A. fumigatus* [21]. To test whether CpcB is associated with production of GT, we examined GT levels in WT, *cpcB* null and *cpcB* complemented strains. As shown in Fig. 5B, whereas WT and complemented strains all produced detectable amount of GT, none of the three *cpcB* null mutants exhibited the GT production. These results indicated that, like SfaD, CpcB is required for proper production of GT.

### CpcB is Needed for Vegetative Growth in the Absence of FlbA or RgsA

**Figure 6:**
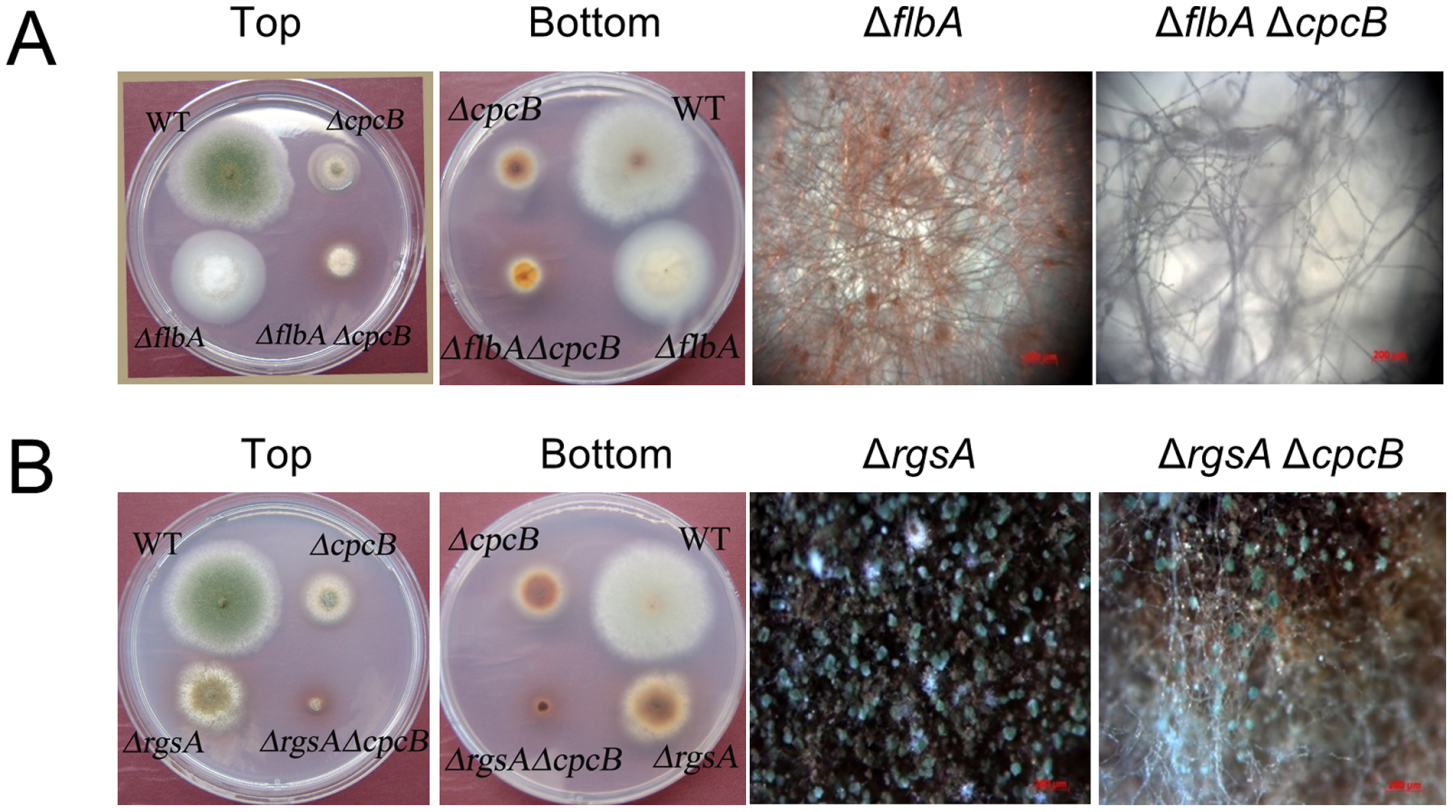
Phenotype of the Δ*flbA*Δ*cpcB* and Δ*rgsA*Δ*cpcB* mutants. Photographs of colonies of WT (FGSC4), Δ*cpcB* (RJMP1.59-8), Δ*flbA* (QK1), and Δ*flbA*Δ*cpcB* (QK3) strains (A); and Δ*rgsA* (QK2) and Δ*rgsA*Δ*cpcB* (QK4) strains (B) grown on solid MMG for 3 days are shown. The two left panels show the point-inoculated strains (top and bottom), and the two right panels show close-up views of single and double mutant colonies shown in the left panels. Photomicrographs were taken by Zeiss Axioplan 2 stereomicroscope. Note the highly restricted growth of the double mutants (A and B), and the lack of conidiation in the Δ*flbA* and Δ*flbA* Δ*cpcB* mutant (A).

FlbA is an RGS protein that rapidly turns off growth signaling mediated by FadA and SfaD:GpgA, and the deletion of *flbA* results in hyper-active hyphal proliferation followed by autolysis in *A. nidulans* (see Introduction). We asked whether CpcB is also associated with FadA-mediated growth signaling as a non-canonical Gβ subunit. This question was addressed by generating the Δ*flbA* Δ*cpcB* double mutant (QK3) and comparing the resulting phenotypes with the Δ*flbA* single mutant (QK1, Table 1). As shown in Fig. 6A, the Δ*flbA* Δ*cpcB* double mutant exhibits severely impaired colony growth and the lack of autolysis even at 7 days (data not shown). These data indicate that CpcB is necessary for proper vegetative growth even in the absence of the key negative regulator FlbA of growth signaling, and implying that CpcB-mediated proliferation control might be independent of FadA. This is further supported by the fact that the Δ*rgsA* Δ*cpcB* mutant displayed highly restricted colony growth, produced fewer conidia with enhanced pigmentation (Fig. 6B). Moreover, different from the Δ*flbA* Δ*sfaD* and the Δ*flbA* Δ*gpgA* mutants, the Δ*flbA* Δ*cpcB* double mutant failed to produce abundant conidiophores. This is consistent with our finding that CpcB is required for proper conidiation in *Aspergillus*, whereas SfaD negatively regulates conidiation [11]. These results indicate CpcB is a critical component of vegetative growth and development, likely independent of FadA and GanB signaling.

## Discussion

In ascomycetous fungi, the biosynthesis of amino acids or charged tRNAs are coordinately controlled by a complex transcriptional network called ‘cross-pathway control’ [53], [54]. The amino acid starvation signal is perceived by the cross pathway regulatory network and results in increased expression of a transcriptional activator encoded by a c-Jun homologue, CpcA, GCN-4, cpc-1 [55]. In the presence of amino acids, the RACK1 homolog CpcB is required to repress this network. The *A. nidulans* CpcB is shown to function in repressing expression of *argB*, and progression and completion of sexual fruiting.

In this study, we further characterized CpcB’s role in governing growth and development in two *Aspergillus* species. Similar to Gib2, CpcB contains the seven WD-40 repeat motif and is predicted to form a seven-bladed β propeller structure characteristic of β transducins [22]. Whereas *C. neoformans* Gib2 is known to interact with two Gγ subunit homologs, Gpg1 and Gpg2, similar to the conventional Gβ subunit Gpb1 [22], *Aspergillus* genomes contain only one probable Gγ GpgA, suggesting that CpcB might interact with GpgA only.

Our central hypothesis for the present study was that CpcB plays a crucial role in governing vegetative growth, spore germination and development in *A. nidulans* and *A. fumigatus*. In *A. nidulans*, while radial growth rates of the *sfaD* and *gpgA* deletion mutants of *A*. *nidulans* grown on solid medium were similar to those observed for WT strains [11], [13], those of the Δ*cpcB* mutant were severely impaired resulting in extremely small colonies (Figs. 2A & 3A), which resembles the mutant lacking the putative GEF RicA [56]. In contrast, in *A. fumigatus*, whereas the *cpcB* deletion mutant exhibited radial growth that is about 80% WT, the Δ*AfusfaD* and Δ*AfugpgA* mutants showed extremely impaired hyphal growth on solid medium (Fig. 3A & B; [21]). These suggest that, despite the very high level of amino acid identity of CpcB and SfaD between the two species, the primary signaling element for hyphal growth can be different. However, in both species, conidial germination has been hampered by the absence of CpcB or SfaD (Figs. 4A & B; [10], [21]), suggesting that both the canonical Gβ SfaD and RACK1 homolog CpcB are required for proper conidial germination.

Our previous studies demonstrated that the developmental activator BrlA is essential for conidiation in *A. nidulans* and *A. fumigates* [19]. Our present study indicates that developmental input(s) from CpcB is required for the proper expression of *brlA* and the transition from vegetative growth to development, as the deletion of *cpcB* results in delayed (∼12∼36 h) and reduced accumulation of *brlA* transcript during asexual developmental progression in both species (Fig. 2C). Furthermore, Northern blot analyses reveal that the absence of CpcB results in severely impaired expression of *abaA*, *wetA*, and *vosA* during asexual and sexual developmental progression in *A. nidulans* (Fig. S1). Consistent with the previous study [24], we also found that CpcB is required for specific steps in sexual development in *A. nidulans*, even in the presence of *veA* ^+^. As shown in Fig. 3A∼D, the deletion of *cpcB* caused highly a reduced formation of cleistothecia under the air-limited and dark conditions that favor sexual development. Moreover, whereas the partially defective *veA1* allele caused the formation of micro- cleistothecia [24], the presence of the WT *veA* allele partially restored the size, but not the number, of cleistothecia (Fig. 3C). Importantly, the *cpcB* null cleistothecia were highly fragile with immature cell walls (reduced pigmentation), and contained no ascospores regardless of the *veA1* or *veA*+ allele, indicating that CpcB plays an essential role in ascosporogenesis and the maturation of cleistothecia. We previously reported that SfaD was essential for the formation of cleistothecia and negative control of Hülle cell formation, as the *sfaD* or *gpgA* null mutant failed to produce any cleistothecia, but produced an excessive number of Hülle cells [11], [13]. The defective sexual fruiting was semi-dominant as the *sfaD* or *gpgA* null mutation affected cleistothecia formation even in outcrosses with WT strains. These suggest that the canonical Gβ SfaD and CpcB may function at different steps of sexual development, yet they both are required for proper ascosporogenesis in *A. nidulans*.

CpcB appeared to be necessary for proper production of ST, but not expression of *stc* genes in *A. nidulans* (Fig. 5A & 5C). In fact, the deletion of *cpcB* resulted in delayed but elevated accumulation of *aflR* and *stcU*. This is quite different from what was observed for the *sfaD*, *gpgA* and *phnA* deletion mutants, where they all failed to produce ST and express *aflR* and *stc* biosynthetic genes [14]. The fact that the overexpression of *aflR* in the *sfaD* deletion mutant was sufficient to restore ST production indicated that SfaD-associated signaling is needed for proper expression of AflR, the key transcriptional activator of *stc* genes [6]. It is our interpretation that the reduced ST production caused by the absence of *cpcB* is likely due to the impaired vegetative proliferation, and unlike SfaD, CpcB does not play a key role in ST biosynthesis.

The Δ*flbA* Δ*cpcB* and the Δ*rgsA* Δ*cpcB* double mutants exhibited enhanced defects in growth and development in *A. nidulans*, which makes it difficult to devise the genetic relationship among CpcB and known heterotrimeric G protein signaling pathways. The simplest explanation for these double mutant phenotypes would be that CpcB is a primary component for vegetative growth, and the non-attenuated (hyper-active) FadA-SfaD:GpgA or GanB-SfaD:GpgA signaling pathways, in the absence of CpcB, would result in drastically altered upstream signaling, which in turn results in extremely hampered growth and development. It was suggested that Gib2 likely stabilizes Gpa1 and facilitates its oscillation between ON and OFF status, and regulates cAMP signaling in *C. neoformans* [22]. Likewise, CpcB might be necessary for the stabilization of one or more of Gα proteins in *Aspergillus*, and a prolonged activation of FadA and GanB by the removal of the cognate negative regulator and the absence of CpcB may cause effects similar to a total lack of heterotrimeric G protein signaling. In this regard, it is noteworthy that the deletion of the putative GEF RicA caused severely impaired growth and the total absence of conidiation in *A. nidulans* [56].

**Figure 7:**
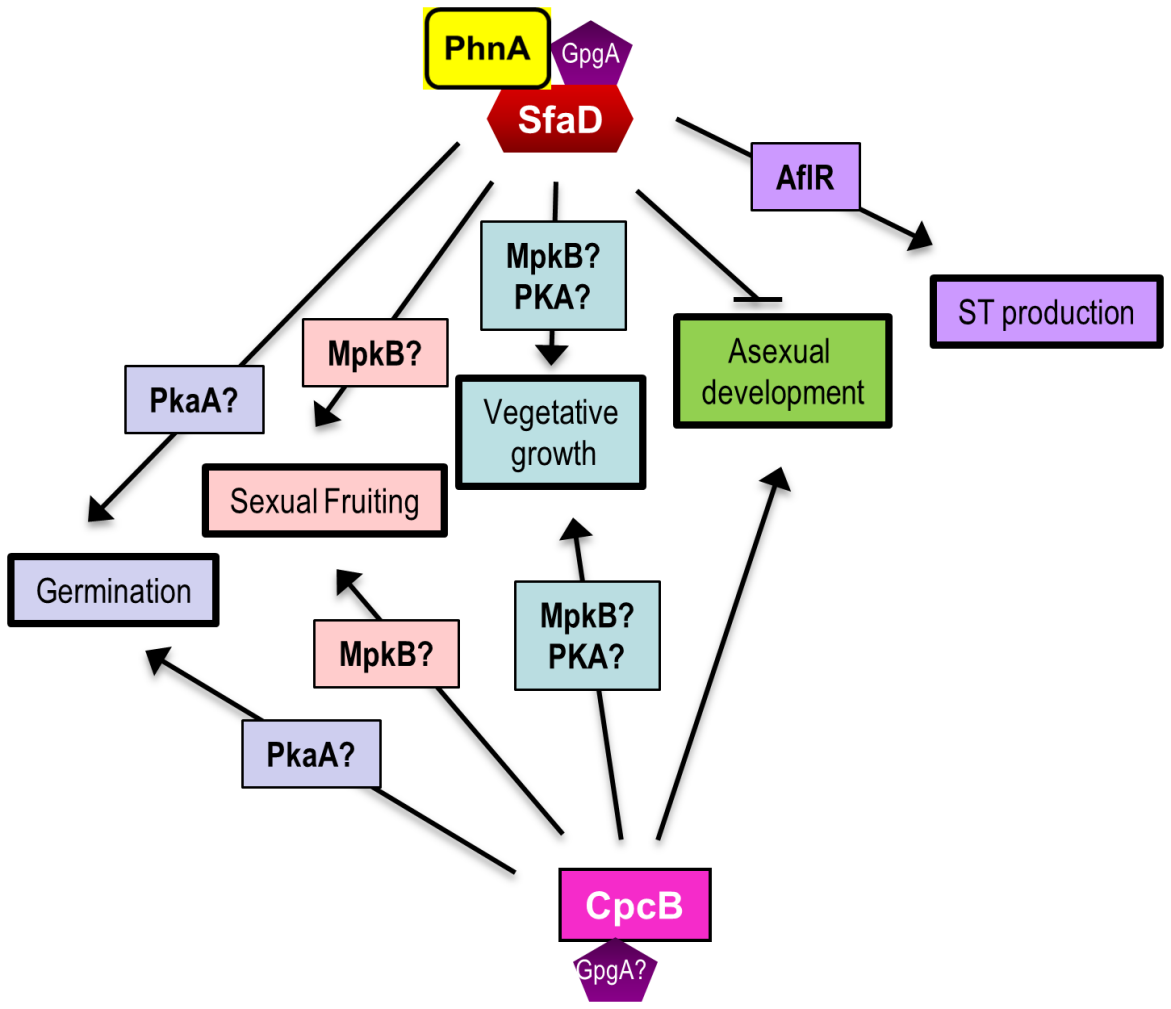
A speculative model summarizing the roles of CpcB and SfaD in governing various biological processes in *A. nidulans*.

Without information on the potential downstream targets of CpcB, it is premature to devise the signal transduction cascade(s) associated with CpcB. Previously, we speculated that MAP kinase(s) might be involved in transducing SfaD:GpgA-mediated signals for sexual reproduction [13], [14]. In accordance with this hypothesis, a recent study revealed that the MAP kinase MpkB is required for sexual fruiting. The deletion of *mpkB* resulted in pleiotropic effects including the lack of cleistothecia formation under any induction conditions for sexual development, increased Hülle cell production, reduced hyphal growth and aberrant conidiophore morphology [57]. These phenotypes are almost identical to those caused by the absence of *sfaD* or *cpcB*. These led us to speculate that SfaD and CpcB mediated signaling might be in part transduced through MpkB. The other key downstream components of vegetative growth and developmental regulation are cAMP-dependent protein kinases (PKA), PkaA and PkaB [3]. PkaA is the primary PKA that positively functions in vegetative growth and spore germination, but negatively controls conidiation and ST production [3], [14]. Whereas the deletion of *pkaB* alone does not cause apparent phenotypic changes, the absence of both *pkaB* and *pkaA* is lethal, i.e., PkaB and PkaA are essential for viability of *A. nidulans*. Overexpression of *pkaB* enhances hyphal proliferation and rescues the growth defects caused by Δ*pkaA*, indicating that PkaB plays a positive role in vegetative growth signaling [58]. Lafon et al. [10] also showed that GanB-SfaD::GpgA-mediated glucose sensing and germination signaling is through PkaA. Collectively, we speculate that CpcB and SfaD mediated signaling for vegetative growth may involve both MpkB and PKA, whereas their signaling for sexual development might be primarily associated with MpkB. A speculative model summarizing the potential roles of CpcB and SfaD in governing various biological processes in *A. nidulans* is presented (Fig. 7).

## Supporting Information

Figure S1 Northern blot analyses for levels of *brlA*, *abaA*, *wetA*, *vosA*, *nsdD* and *veA* transcripts upon asexual and sexual developmental induction of WT (FGSC4) and Δ*cpcB* (RJMP1.59-8) strains of *A. nidulans.* C indicates conidia, and the numbers indicate time post developmental induction. (TIF)

Table S1 Oligonucleotides used in this study. (DOCX)
