# Supplementary figures and images for: Gβ-Like CpcB Plays a Crucial Role for Growth and Development of Aspergillus nidulans and Aspergillus fumigatus
Source: PLoS One. 2013 Jul 30;8(7):e70355. doi: 10.1371/journal.pone.0070355 (PMC3728086; doi:10.1371/journal.pone.0070355)

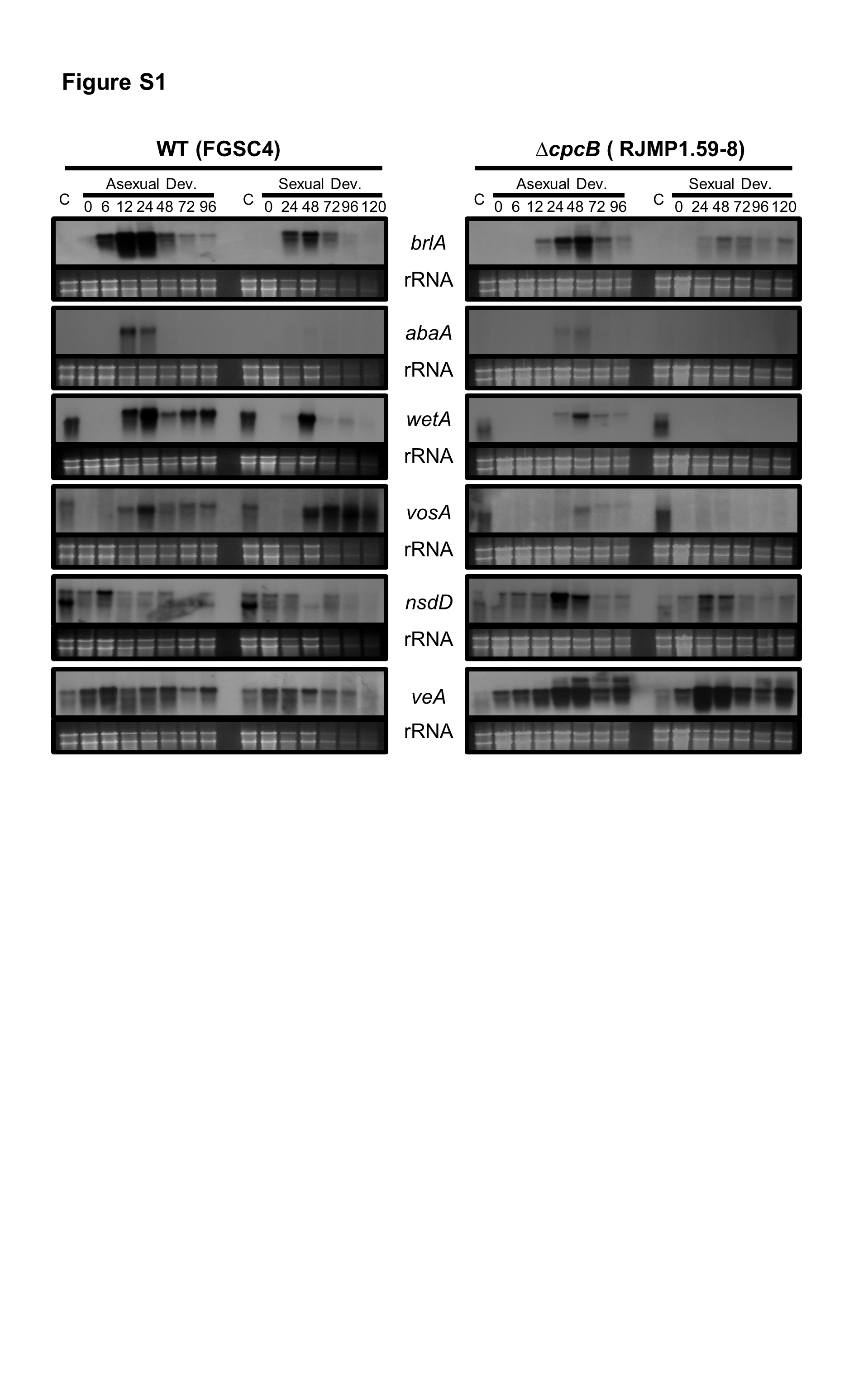

Supplement: Figure S1 — (TIF) [file pone.0070355.s001.tif]
