# Supplementary material for: Gβ-Like CpcB Plays a Crucial Role for Growth and Development of Aspergillus nidulans and Aspergillus fumigatus
Source: PLoS One. 2013 Jul 30;8(7):e70355. doi: 10.1371/journal.pone.0070355 (PMC3728086; doi:10.1371/journal.pone.0070355)
Supplement: Table S1 — (DOCX) [file pone.0070355.s002.docx]

**Kong et al., Table S1. Oligonucleotides used in this study.**

| **Name** | **Sequence (5’→3’)** | **Purpose** |
| --- | --- | --- |
| oJH-83 | GGATGTATCGTGACTGGCCTTCGG | *AfupyrG* 5'-For |
| oJH-86 | TAATTCGCGGCATACGGTGTCTAA | *AfupyrG* 3'-Rev |
| oPX-105 | AGTTAATCTCTGGCCCTCTTTGTC | *Ani-cpcB* 5' For |
| oPX-106 | GGTGAAGAGCATTGTTTGAGGCA TCACGGCGTTCGAAGATGGAGG | *Ani-cpcB* 5' Rev with *Af-pyrG* tail |
| oPX-107 | AGTGCCTCCTCTCAGACAGAATA AGGACTTGAGAGCGAATGGGTTG | *Ani-cpcB* 3' For with *Af-pyrG* tail |
| oPX-108 | TACCGACAAAGCACTGGTCTTCC | *Ani-cpcB* 3' Rev |
| oPX-109 | AGCGAGGTATTCTCGAGTCCGAC | *Ani-cpcB* 5' For Nest |
| oPX-110 | TCCTGCGTGCTTTAGAACTACTCC | *Ani-cpcB* 5' Rev Nest |
| oLW-1 | ATATGGTACCACTACACGGGAGGCAGACAGC | *Ani-upCpcB*5’For w/ KpnI |
| oLW-2 | ATATGAATTCGTCTTCCCCTCAGCAACCCCA | *Ani-CpcBdown*3’Rev w/EcoRI |
| oLW-12 | GAGAAGAAGAGCAAGGTCGA | *Ani-CpcB*-Complement5’ |
| oLW-13 | CTTTATAGTCCTCGAGAGCG | *Ani-CpcB*-Complement3’ |
| oLW-16 | CAAGCTCAAGCGCTAGCTCTTG | seq01R |
| oLW-17 | GAGGTGGCTTGAACAGAATGCC | seq02F |
| oLW-19 | GACCCTGATCATCTGGAACCTG | seq03F |
| oLW-21 | CATGTTCTCTCGCTTGCTGCAC | seq04F |
| oLW-23 | GGCCAGACTCTTTTCGCTGGTT | seq05F |
| oLW-25 | CAGTACAACAGCCAGCGAAGAG | seq06F |
| oHS-350 | ACAGGTTTCCCGACTGGAAAGC | pHS-8 Sequence-F |
| oHS-351 | AAGGCACTCTTTGCTGCTTGGG | pHS-8 Sequence-R |
| oPX-111 | TGGTCCTGAAACTAATAGAGTGAC | *AfucpcB* 5' For |
| oPX-112 | TTTGTAGGCTTTGGGCTGTTCACAA  TAAGCCCTAAACTAGCCCGTTCTG | *AfucpcB* 5' Rev w/*AnipyrG* tail |
| oPX-113 | CTGATCTACCCCTTGGAACGCAGCA  TCCGACATGTCTAGCTTGATTCG | *AfucpcB* 3' For w/*AnipyrG* tail |
| oPX-114 | TGAACCTATCCGAGATTGCCACTG | *AfucpcB* 3' Rev |
| oPX-115 | AGAACAGCTCCAGGAGTACGACC | *AfucpcB* 5' For Nest |
| oPX-116 | TACGCACAGACGAAGGCAGCAC | *AfucpcB* 3' Rev Nest |
| oLW-3 | GTCAAGGCCCACCTGCGCATT | *Hygr*5’For |
| oLW-9 | CGCAGTCAGGCACCGTGTATG | *Hygr*3’ |
| oLW-10 | GATTTCATACACGGTGCCTGACTGC  GGGTCCTGAAACTAATAGAGTGACC | tail*Afu-upCpcB*5’ |
| oLW-4 | GCAAACCTCTGCAGAGCGGTC | *Hygr*5’ForNest |
| oLW-7 | GACCGGCAACCCCAAGGACAT | *CpcB*3’RevNest |
| oLW-11 | GAGAAGAACGAGCTCGCCATGCC | *Afu-downCpcB*3’Nest |
| oNK-412 | TCACATCTCGATGATTGGTTGAATG | *Ani-flbA*-DF |
| oNK-413 | ACTTCTGCAGTCGGAATTGGCCTG TGGCATTGAAGAGTGCAGGTCGGAG | *AniflbA*-IR with *AnipyroA* tail |
| oNK-414 | TGGTGAGAACACATGCACAACTTG ACAGTAATTATCTACACGCGTGATG | *AniflbA*-IF with *AnipyroA* tail |
| oNK-415 | ACTACTCACTACCTAACTTGACTG | *AniflbA*-DR |
| oNK-416 | TGGTTGAATGGTGTATGGGTCAGC | *AniflbA*-NF |
| oNK-417 | TGTAGCTTTCGTTCAGGCGATAGTG | *AniflbA*-NR |
| oNK-540 | TGTTCCTGGATGATAGTCTTCTAG | *Ani5755*-DF for *rgsA* |
| oNK-541 | ACTTCTGCAGTCGGAATTGGCCTG TTGACTGAACGTATCTTAAGAGTG | *Ani5755*-IR with *pyroA* tail |
| oNK-542 | TGGTGAGAACACATGCACAACTTG ACGTCGTGGCACAATCTTCCGGTG | *Ani5755*-IF with *pyroA* tail |
| oNK-543 | AGCTCCAATACACTGCATGTGGAG | *Ani5755*-DR for *rgsA* |
| oNK-544 | TGTCGCCGACCACTACTACTACC | *Ani5755*-NF for *rgsA* |
| oNK-545 | TTGAACTATCTTAACGAGGGTGAC | *Ani5755*-NR for *rgsA* |
| oBS-08 | GCAATGTAAAGCTAACGTGCGTG | 5’*AnipyrG* marker |
| oBS-09 | TGCCTTTAAGCTTCGGGTAGAG | 3’*AnipyrG* marker |
| oNK-395 | ATCTCATGGGTGCTGTGCGAAAGG | 5’*AnipyroA* marker |
| oNK-396 | TTGCATCGCATAGCATTGCATTGC | 3’*AnipyroA* marker |
| oNK-269 | ATGCGAAATCAGTCCAGCCTGTC | 5’*AnibrlA* probe |
| oNK-270 | TCATCCCAGCCGTCCAGGCTCAT | 3’*AnibrlA* probe |
| oNK-611 | AGGTTAAGCCGCCATTTGAGTCATC | 5’*AniabaA* probe |
| oHS-159 | AATTCTGCAGGACAGCCTCAACCGCAGTATGTTC | 3’*AniabaA* probe |
| oJA150 | CAGTACGTCAATATGGAC | 5’*AniwetA* probe |
| oJA151 | GTGAAGTTGACAAACGAC | 3’*AniwetA* probe |
| oNK-14 | ATATGAATTCATGAGTGCGGCGAACTATCCAG | 5’*AnivosA* probe |
| oNK-15 | ATATGTCGACTCACCGAGGAGTTCCGTTCGCTG | 3’*AnivosA* probe |
| oNK-936 | ATATAAGCTTATGGGATCACTAGAGGCTGGACATAG | 5’*AninsdD* probe |
| oNK-937 | ATATGCGGCCGCATGACTCCTCGGTGACACCGAGTC | 3’*AninsdD* probe |
| oNK-11 | ATATAAGCTTAATGGCTACACTTGCAGCACCAC | 5’*AniveA* probe |
| oNK-12 | ATATGTCGACTTAACGCATGGTGGCAGGCTTTG | 3’*AniveA* probe |
| oNK-962 | TCCGATAATTACCGTCTCGATGG | 5’*AnistcU* probe |
| oNK-963 | TCCACTGATCCATTCGGCCGCATC | 3’*AnistcU* probe |
| oNK-964 | AGCGATCAGCCAGCAATCCACAC | 5’*AniaflR* probe |
| oNK-965 | AATCGTCTTCTAGATGGACCAGG | 3’*AniaflR* probe |
| oNK-594 | TGAGATCCCAGGGTAATATGTCTG | 5’*AfubrlA* probe |
| oNK-595 | TACTCATCCCATTCCATACTGATC | 3’*AfubrlA* probe |
| oLT-3 | ATATGGTACCATGGCTACTGACTGGCAGCCCGAG | 5’*AfuabaA* probe |
| oLT-4 | ATATGCGGCCGCTTGGACCGCCTCAGTTGCATTGG | 3’*AfuabaA* probe |
| oJH-24 | CCATACTTTGTTCCGCAG | 5’*AfuwetA* probe |
| oJH-29 | AACGCCCAAACGACCATT | 3’*AfuwetA* probe |
